# Normal inter-limb differences during the straight leg raise neurodynamic test: a cross sectional study

**DOI:** 10.1186/1471-2474-13-245

**Published:** 2012-12-10

**Authors:** Benjamin S Boyd, Philip S Villa

**Affiliations:** 1 Department of Physical Therapy, Samuel Merritt University, 450 30th Street, Oakland, CA, 94609, USA; 2 Kaiser Permanente, 975 Sereno Drive, Vallejo, CA, 94589, USA

## Abstract

**Background:**

The straight leg raise (SLR) neurodynamic test is commonly used to examine the sensitivity of the lower quarter nervous system to movement. Range of motion during the SLR varies considerably, due to factors such as age, sex and activity level. Knowing intra-individual, inter-limb differences may provide a normative measure that is not influenced by such demographic characteristics. This study aimed to determine normal asymmetries between limbs in healthy, asymptomatic individuals during SLR testing and the relationship of various demographic characteristics.

**Methods:**

The limb elevation angle was measured using an inclinometer during SLR neurodynamic testing that involved pre-positioning the ankle in plantar flexion (PF/SLR) and neutral dorsiflexion (DF/SLR). Phase 1 of the study included 20 participants where the ankle was positioned using an ankle brace replicating research testing conditions. Phase 2 included 20 additional participants where the ankle was manually positioned to replicate clinical testing conditions.

**Results:**

The group average range of motion during PF/SLR was 57.1 degrees (SD: 16.8 degrees) on the left and 56.7 degrees (SD: 17.2 degrees) on the right while during DF/SLR the group average was 48.5 degrees (SD: 16.1 degrees) on the left and 48.9 degrees (SD: 16.4 degrees) on the right. The range of motion during SLR was moderately correlated to weight (−0.40 to −0.52), body mass index (−0.41 to −0.52), sex (0.40 to 0.42) and self-reported activity level (0.50 to 0.57). Intra-individual differences between limbs for range of motion during PF/SLR averaged 5.0 degrees (SD: 3.5 degrees) (95% CI: 3.8 degrees, 6.1 degrees) and during DF/SLR averaged 4.1 degrees (SD: 3.2 degrees) (95% CI: 3.1 degrees, 5.1 degrees) but were not correlated with any demographic characteristic. There were no significant differences between Phase 1 and Phase 2.

**Conclusions:**

Overall range of motion during SLR was related to sex, weight, BMI and activity level, which is likely reflected in the high variability documented. We can be 95% confident that inter-limb differences during SLR neurodynamic testing fall below 11 degrees in 90% of the general population of healthy individuals. In addition, inter-limb differences were not affected by demographic factors and thus may be a more valuable comparison for test interpretation.

## Background

The straight leg raise (SLR) is a common neurodynamic test used to examine the mechanosensitivity of the lower extremity nervous system in individuals with low back or lower extremity pain [1-4]. Structural differentiation is necessary to determine if symptom provocation and range of motion restrictions are related to neural tissue [5]. Pre-positioning in ankle dorsiflexion compared to plantar flexion is commonly utilized for purposes of structural differentiation during SLR testing [1,2,6,7] and distinguishes the SLR neurodynamic test from a hamstring muscle length test [8]. From here forward SLR will refer to neurodynamic testing. It has been proposed that identification of a “positive,” clinically relevant test should include consideration of three components [5]. These components include 1) reproduction of the patient’s symptoms in whole or in part, 2) distant movements away from that region altering the symptoms (structural differentiation), and 3) identification of differences in sensory, range of motion or resistance to movement noted between limbs or known norms [5]. Limb elevation angle at the point of a sensory response provides a mobility measurement for the third component. Ideally, normative SLR range of motion in healthy, asymptomatic individuals could be used for comparisons to testing in clinical populations. Unfortunately, when used as a neurodynamic test, normal SLR range of motion is highly variable, averaging from 40° to 85° [1,3,6,9]. The large degree of variability in range of motion makes valid identification of mobility impairments difficult.

Previous literature has demonstrated that lower extremity range of motion is highly dependent upon multiple factors, such as age, [10-12] sex, [8,10,11] and limb dominance [13]. These demographic factors may explain much of the variability in SLR range of motion but this has yet to be investigated. If these relationships do exist, establishing normative SLR range of motion becomes quite problematic. An alternative approach is to look at symmetry of SLR range of motion within individuals. Previous literature of healthy, asymptomatic individuals found significant intra-individual asymmetries in isolated ankle motions [14] as well as differences between limbs during upper limb neurodynamic testing [15]**.** Intra-individual, inter-limb differences may be a more useful measure for establishing normative values for SLR testing, as they are less likely to be influenced by other factors such as age, sex, weight and activity level. Providing evidence of normal inter-limb differences during SLR testing in the healthy, asymptomatic population will allow for future comparisons of the differences between the affected limb and unaffected limb in patients experiencing unilateral pain.

The primary aims of this study were to; 1) determine the relationship between demographic characteristics and overall SLR range of motion, 2) quantify inter-limb differences during SLR testing in healthy, asymptomatic individuals, under both research conditions and clinical testing conditions. Normal overall SLR range of motion and inter-limb differences are presented with correlations to various demographic characteristics and implications for test interpretation.

## Methods

This cross sectional study included two phases involving SLR neurodynamic testing performed where the ankle was positioned using an ankle brace to replicate research testing conditions (Phase 1) and where the ankle was manually positioned to replicate clinical testing conditions (Phase 2). Each phase included a unique set of 20 healthy, asymptomatic participants (n=40 total). Participants from Phase 1 were previously reported for purposes of validating the measurement device but data related to inter-limb difference has not been previously published [7].

Participants were recruited from local academic and medical facilities. Inclusion criteria included minimum flexibility requirements of isolated ankle range of motion >0° dorsiflexion and >30° plantar flexion, full knee extension, and hip flexion >90° with the knee flexed. Exclusion criteria included current or recent (> 3 consecutive days in past 6 months) low back or lower extremity pain, peripheral neuropathy, diabetes mellitus, complex regional pain syndrome, chemical dependence or alcohol abuse, a history of lower extremity nerve trauma, lumbar spine surgeries, or chemotherapy use. The Samuel Merritt University Institutional Review Board approved this study and assured ethical treatment of participants. Prior to testing, written informed consent was obtained. Prior to SLR testing each participant completed the Modified Baecke Questionnaire (MBQ), which is a self-report measure on activity level [16].

### Neurological testing

In order to rule out potential sub-clinical injuries to the nervous system, a segmental neurological examination was performed to confirm that the participants had no signs of conduction loss. In brief, dermatome testing with a 10 gram monofilament was performed in bilateral sensory distributions for segments L3 (medial knee), L4 (medial ankle), L5 (dorsum of foot), S1 (lateral heel) and graded as present or absent. Myotome testing was performed against manual resistance for segments L3 (quadriceps), L4 (tibialis anterior), L5 (extensor hallucis longus), and S1 (fibularis longus and brevis) and graded as normal, mild/moderate weakness, severe weakness, or absent. Deep tendon reflexes were performed for L4 (patellar tendon), L5 (semitendinosis tendon), S1 (Achilles tendon) and graded as present or absent. Quantitative sensory testing included vibration perception thresholds (VPT) in bilateral halluces (distal pad) using a 60 Hz Biothesiometer (Bio-Medical Instruments Company, Newbury, OH, USA) with a scale of 0–50 V. Participants were instructed to indicate the first moment when the vibration was felt as it was slowly turned up from zero and VPT is reported as an average voltage (two trials each limb). Previous literature has identified normal ranges for VPT testing at the halluces as 15V or lower [17,18].

### SLR testing

Participants in both phases of testing were placed in a standardized start position which included lying supine on a plinth with a 2.5 cm thick foam head support. They were positioned with their spine in neutral within the coronal plane with their upper arms resting at their sides. Their lower limbs were positioned in neutral abduction. A hand-held inclinometer was placed against the anterior aspect of the mid-tibia. Measuring limb elevation angle with this device demonstrates excellent reliability (ICC: 0.95-0.98), validity (ICC: 0.88-0.99) and standard error of measurement (0.54-1.22°) when used during the SLR test [7]. The ankle was placed in either dorsiflexion or plantar flexion prior to performing the hip flexion component of the SLR. For phase 1, the ankle was secured in 0º dorsiflexion or 30º plantar flexion using an ankle brace and straps (Anatomical Concepts, Inc., Youngstown, OH) [1,2]. For phase 2, the ankle was manually placed in dorsiflexion or plantar flexion to the point of firm resistance as felt by the examiner, as is commonly done clinically. Ankle position was monitored in this phase by use of a twin-axis electrogoniometer (Noraxon, USA, Scottsdale, AZ) that was placed laterally across the ankle with the proximal end parallel to the fibula and the distal end parallel to the 5^th^ metatarsal [3]. The goniometer was held in place with double-sided tape and straps. Dorsiflexion to 0° was utilized due to the frequency of limitations in ankle dorsiflexion range when the knee is in full extension [19]. Using an electronic trigger held in their dominant hand resting on their abdomen, the participant was instructed to indicate when any sensory response was elicited during the SLR test. The electrogoniometer and hand-held trigger data were acquired at 1000 Hz using a Myosystem 1400 unit (Noraxon, USA, Scottsdale, AZ).

The SLR consisted of placing the knee in end range extension, determined by the examiner as end range resistance (R2), followed by bringing the limb into hip flexion. Care was taken to avoid movement of the limb in the transverse or coronal planes. The movement was stopped at the first moment any sensory response was indicated, including but not limited to the sensation of stretch, pulling, tension, pain, numbness, or tingling. The use of healthy participants without nerve injury meant that true “positive” neurodynamic test findings (as outlined above) were not possible in this study as there are no “symptoms” to be provoked. However, mobility limitations due to neurogenic sensory responses are common in healthy individuals during neurodynamic testing and should not be interpreted as pathological [15]. Therefore, this study sought to replicate clinical testing procedures by comparing symmetry of range of motion during the two SLR variations taken to the first onset of any sensory response to assist with comparisons to patients with neuropathic pain. The pelvis and lumbar spine were not stabilized to match clinical testing procedures and as these motions, in addition to hip flexion, theoretically contribute to increasing stress on the posterior neural structures of the lower quarter [7]. The limb elevation angle was measured at this point and then the limb was returned to a resting position on the mat [7]. The SLR was performed twice with the ankle in dorsiflexion (DF/SLR) and twice with the ankle in plantar flexion (PF/SLR) with the order randomized to negate the effect of repeated testing. All tests were performed by one examiner with over nine years of clinical and research experience in neurodynamic testing.

### Statistical analysis

The mean of both trials for overall range of motion and inter-limb difference (defined as the absolute difference between right and left limbs) were utilized for statistical analyses using IBM SPSS Statistics, version 19 (IBM Corporation, Somer, NY). Reliability between the two trials was assessed using Intraclass Correlation Coefficient (ICC_2,1_) calculations with 95% confidence intervals and 95% limits of agreement [20]. Limits of agreement provide the range within which the expected difference between two repeated measurements within an individual would fall 95% of the time [20]. Repeated measures analysis of variance (ANOVA) was utilized to compare range of motion differences between limbs and testing conditions. Comparisons were made between Phase 1 and 2 using independent samples t-tests. Correlation coefficients were used to determine relationships between demographic characteristics and range of motion measures. The strength of correlations were determined as 0.00 to 0.25 = little to no relationship, 0.26 to 0.50 = fair degree of relationship, 0.51 to 0.75 = moderate to good relationship, and 0.76 to 1.00 = good to excellent relationship [21]. Power analysis revealed that with 40 participants and an alpha of 0.05, we would have 80% power of detecting a correlation of 0.37 which represents at least a “fair” relationship. We did not have adequate power to detect interaction effects. For purposes of generalizing from this sample to the greater population, the upper limit of a tolerance interval (one sided test) was calculated in order to identify a threshold within which 90% of the values for “normal” inter-limb differences can be expected in the population with 95% certainty [22]. Alpha was set at 0.05.

## Results

The demographic characteristics of participants are presented in Table 1. The participants in Phase 1 were slightly older by an average of seven years and rated themselves as slightly more active on the MBQ.

**Table 1:** Participant Demographics and correlations

	**Subgroups**		**Combined groups**	**Correlations (compared to combined groups)**					
				**PF/SLR range of motion**			**DF/SLR range of motion**		
	Phase 1	Phase 2	Both Phases	Left	Right	Inter limb difference	Left	Right	Inter limb difference
Age (years)	36.9 ± 12.8 *	29.4 ± 7.3 *	33.1 ± 11.0	0.18	0.23	0.02	0.21	0.28	-0.14
Height (m)	1.7 ± 0.1	1.7 ± 0.1	1.7 ± 0.1	-0.18	-0.15	-0.25	-0.14	-0.09	-0.04
Weight (kg)	69.9 ± 15.9	68.3 ± 15.1	69.1 ±15.3	-0.47 †	-0.52 †	-0.18	-0.42 †	-0.40 †	-0.04
BMI (kg/m^2^)	24.9 ± 4.4	25.1 ± 5.5	25.0 ± 4.9	-0.45 †	-0.52 †	-0.09	-0.41 †	-0.42 †	-0.05
Sex (% female)	85%	70%	77.5%	0.40 †	0.42 †	0.06	0.42 †	0. 40 †	-0.09
Hand dominance (% right)	70%	80%	75%	-0.22	-0.27	-0.04	-0.23	-0.23	-0.05
VPT (V)	7.8 ± 3.1	6.4 ± 1.9	7.1 ± 2.6	0.06	0.11	0.02	0.13	0.18	-0.06
MBQ (total score)	9.5 ± 1.2 *	8.6 ± 1.5 *	9.0 ± 1.4	0.50 †	0.57 †	0.14	0.51 †	0.52 †	0.18

*BMI* = body mass index.

*VPT* = vibration perception threshold at distal pad of halluces; 0-50V scale with higher numbers indicating poorer sensation and lower numbers indicating better sensation and normal being between 0-15V.

*MBQ* = Modified Baecke Questionnaire; 3-15 point scale with higher numbers indicating higher self-reported activity levels and lower numbers indicating lower self-reported activity levels.

*PF* = plantar flexion.

*DF* = dorsiflexion.

*SLR* = straight leg raise.

* = statistically significant difference between groups.

† = statistically significant correlation coefficient (*r*).

Alpha set at 0.05.

### Neurological testing

All participants had intact sensation in all dermatome levels bilaterally with normal and equal strength bilaterally on myotome testing. Deep tendon reflexes were equal bilaterally in all participants. VPT values were equivalent between limbs and averaged 7.1 (2.6 SD) V which is well within normal ranges (<15 V) [17,18].

### Reliability

There was no significant difference in SLR range of motion and excellent reliability between trials for Phase 1 (p=0.332-0.899; ICC_2,1_: 0.96-0.99) and Phase 2 (p=0.356-0.839; ICC_2,1_: 0.94-0.97) so both groups were combined for the remainder of the reliability analysis. For repeated testing, ICCs_2,1_ were 0.97 (95% CI: 0.94, 0.98) for left PF/SLR with 95% limits of agreement between −10.5° and 9.9°. With right PF/SLR, the ICC was 0.96 (95% CI: 0.93, 0.98) with 95% limits of agreement between −12.0° and 11.1°. For left DF/SLR the ICC was 0.98 (95% CI: 0.96, 0.99) with the 95% limits of agreement between −8.4° and 8.8°. For right DF/SLR the ICC was 0.96 (95% CI: 0.93, 0.98) with the 95% limits of agreement between −9. 8° and 11.9°.

### Ankle positioning during SLR

During Phase 2, there was no difference between the initial ankle position between trials (Table 2) with good reliability (ICC_2,1_: 0.78-0.89), indicating that the ankle was positioned consistently between trials. The total ankle range of motion utilized in Phase 2 (30.0-32.3°) with manual stabilization (Table 2) was similar to that utilized in Phase 1 (30°) with fixation using the brace.

**Table 2:** SLR range of motion

	**PF/SLR**			**DF/SLR**		
	** Left**	** Right**	**p value**	** Left**	** Right**	**p value**
**Hip elevation angle**						
Phase 1	59.7° (18.5°)	60.2° (18.7°)	0.693	51.5° (17.6°)	52.9° (18.3°)	0.219
Phase 2	54.5° (14.8°)	53.3° (15.3°)	0.440	45.6° (14.4°)	44.9° (13.7°)	0.611
Both Phases	57.1° (16.8°)	56.7° (17.2°)	0.752	48.5° (16.1°)	48.9° (16.4°)	0.692
**Ankle position** (Phase 2 only)						
Initial position	33.8° (9.8°) PF	32.0° (12.8°) PF	0.599	2.0° (6.3°) PF	1.1° (6.3°) PF	0.750
Ending position	31.1° (9.5°) PF	30.5° (12.4°) PF	0.821	3.4° (7.0°) PF	0.8° (6.9°) DF	0.224

*PF* = plantar flexion.

*DF* = dorsiflexion.

*SLR* = straight leg raise.

### SLR range of motion

**Figure 1:**
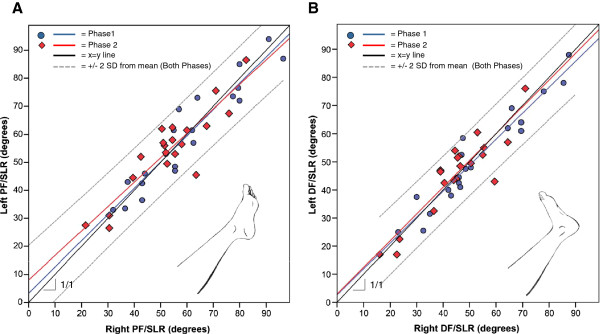
**Overall SLR range of motion.** SLR range of motion during PF/SLR (**A**) and DF/SLR (**B**) for the right (x-axis) and left (y-axis) are presented in degrees for Phase 1 (red) and Phase 2 (blue) including best fit lines for each phase. Black line represents the absolute y = x condition as indicated by the 1/1 slope. The grey dotted lines represent ± 2 standard deviations from the mean for Both Phases. Data points above the y = x line are indicative of more SLR range of motion on the left limb and those below this line indicate more on the right limb.

**Figure 2:**
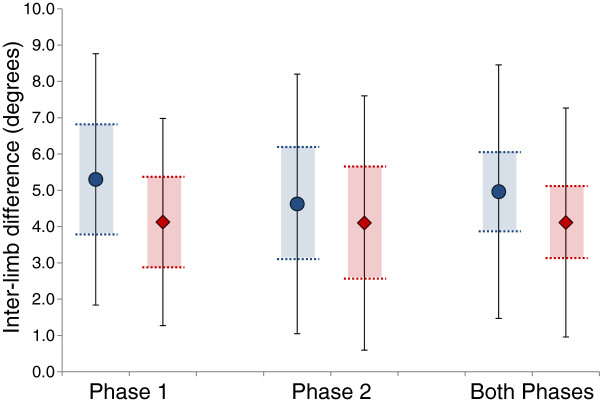
**Intra-individual, inter-limb differences during SLR testing.** Mean inter-limb differences are presented in degrees for Phase 1, Phase 2 and Both Phases for PF/SLR (blue) and DF/SLR (red). Solid black error bars represent standard deviations. Horizontal dotted lines and shaded area represent the 95% confidence interval for the mean inter-limb difference for PF/SLR (blue) and DF/SLR (red).

The SLR range of motion ranged from approximately 15° to over 90° (Figure 1). When examining group means for SLR range of motion, there was no difference between the right and left limbs during either PF/SLR and DF/SLR (Table 2). In fact, the group average of both phases appears nearly identical between limbs (<1° difference). However, there is a significant difference when looking at the average intra-individual, inter-limb differences (Figure 2). For both phases combined, the inter-limb difference with PF/SLR averaged 5.0° (3.5° SD; 95%CI: 3.8°, 6.1°) and 4.1° (3.2° SD; 95%CI: 3.1°, 5.1°) with DF/SLR (Figure 2). Based upon the upper limit of tolerance interval calculations, we can be 95% sure that 90% of the general population would have inter-limb differences of no greater than 10.9° for PF/SLR and 9.4° for DF/SLR.

One participant was an outlier as their inter-limb difference was greater than 3 SDs above the mean during PF/SLR (17.0°) and during DF/SLR (16.5°). Further evaluation of this participant’s data revealed no evidence of confounding variables such as asymmetrical recreational activities, previous injuries or surgeries and confirmed that they were asymptomatic and had a normal neurological exam. For comparison purposes, when this individual was removed from the data analysis, the inter-limb difference was not remarkably different with 4.7° (2.9° SD; 95%CI: 3.7°, 5.6°) during PF/SLR and 3.8° (2.5° SD; 95%CI: 3.0°, 4.6°) during DF/SLR. The upper limit of tolerance interval also was similar with this individual removed; we can be 95% sure that 90% of the general population would have inter-limb differences of no greater than 9.6° for PF/SLR and 8.0° for DF/SLR.

Overall SLR range of motion was moderately correlated with several demographic characteristics of the participants (Table 1). Specifically, bilateral SLR range of motion during both PF/SLR and DF/SLR had a moderate negative correlation with weight and BMI such that higher weight or BMI was associated with less SLR range of motion. As expected, sex was moderately associated with SLR range of motion indicating that females had more SLR range of motion than men. Lastly, there was a moderate positive correlation with self-reported activity level on the MBQ. This indicates that individuals that reported a higher activity level had more SLR range of motion. There were no associations found between the SLR range of motion and age, height, hand dominance or VPT. In contrast, inter-limb difference was not significantly correlated with any demographic characteristic (Table 1).

## Discussion

SLR neurodynamic testing range of motion is highly variable, ranging from approximately 15° to over 90° with a moderate association with multiple demographic characteristics, such as sex, weight, BMI and activity level. Specifically, heavier and less active individuals had lower SLR range of motion bilaterally compared to more active individuals who weighed less, just as women had more SLR range of motion bilaterally compared to men. The correlations between these demographic characteristics and overall SLR range of motion were similar bilaterally suggesting that the influence of these factors is equivalent in each limb. Previous studies have found similar variability in SLR range of motion [1,3,6,9] and that females have more SLR range of motion compared to men [23]. Establishing a cutoff for normal SLR range for motion is problematic with such a high degree of variability and with so many demographic characteristics related to mobility.

In contrast, variability in inter-limb differences was much smaller and was independent of these demographic factors. For purposes of generalizability to the greater population, we can use the upper limit of a tolerance interval. Based upon this calculation, we can be 95% certain that “normal” inter-limb differences would be no greater than 10.9° for PF/SLR and 9.4° for DF/SLR in 90% of the general population of healthy individuals. Findings above these ranges could be considered non-normal and potentially important if found in a patient experiencing unilateral lower extremity pain. Further validation for this threshold comes from two previous studies that examined the inter-limb difference in symptomatic individuals. One study found an average of 12° less mobility on the symptomatic side in people with low back pain with or without lower extremity pain with a positive SLR test [4]. The other study found an average of 30° (SD 10°; range 10° to 55°) less range of motion in people with unilateral lumbar radiculopathy [24].

Utilizing intra-individual, inter-limb differences as the normative standard provides added value because this measurement is independent of various demographic characteristics that commonly impact overall SLR range of motion. In contrast, comparing group means between limbs of healthy, asymptomatic individuals to establish the normative standard for asymmetry in SLR range of motion does not tell the whole story of normal responses to SLR testing. If equal percentages of individuals have greater SLR range of motion on the left (above the y=x line in Figure 1) as do have on the right (below the y=x line in Figure 1), the group averages will equal out and appear to be no different. In fact, we found that considerable intra-individual asymmetries can be present even in healthy, asymptomatic individuals (Figure 2) despite nearly identical group means (Table 2). This is consistent with a previous study where greater than 5° inter-limb differences in ankle range of motion has been documented despite no difference in group mean comparisons [14]. Clinically, intra-individual, inter-limb comparisons are valuable to help determine if neurodynamic involvement is present, which reinforces the need for normative values for this inter-limb difference. Recently, mean inter-limb differences of 7° (6.6° SD) between the dominant and non-dominant limb were documented during upper limb neurodynamic testing [15]. While a threshold level was not presented in this study, one can be calculated from their data using a similar tolerance level upper limit such that we could be 95% certain that 90% of healthy individuals would have no more than a 18.4° inter-limb difference during upper limb neurodynamic testing. This range of “normal” inter-limb differences is higher than in the SLR. We speculate that this difference reflects how asymmetrical use of the upper limbs is more common than for the lower limbs, but further research is necessary to substantiate this hypothesized rationale for the differences noted.

Phase 1 aimed to control the confounding variable of ankle positioning by strict fixation of the ankle position as has been done in previous studies [1-3,6]. It is equally important to test the reliability and validity of manual fixation of ankle positioning during SLR testing, as was the aim of Phase 2. Previous research has suggested that ankle dorsiflexion to 10° with the knee in full extension and during SLR testing is difficult to achieve and dorsiflexion may be limited to only 4.3-4.8° (SDs: 3.6-4.8°) in this position [6,19]. For this reason, a neutral ankle position was targeted with DF/SLR in the present study. Repeatability of ankle positioning had good reliability (ICC_2,1_: 0.78-0.89), but tended to be in 1.1° to 2.0° degrees shy of neutral dorsiflexion at the beginning of testing. On average, the ankle position changed by between 1.4° and 2.7° from the beginning to the end of SLR testing. This suggests that there was a slight shift in ankle position during manual fixation of the ankle, but that the change averaged less than 3° and represents a potential confounding variable that may have influenced the outcome measures. Since there were no significant differences in inter-limb measurements between test phases (Figure 2) and reliability of measuring SLR range of motion was equivalent between phases, the threat to the overall study conclusion is minimal.

The question remains as to why healthy, asymptomatic individuals are not perfectly symmetrical. It is unlikely that sub-clinical nerve injuries are responsible for the asymmetries documented, as all participants had normal lower extremity segmental neurological exams and quantitative sensory testing within normal ranges. Despite considerable efforts to exclude individuals with injuries to the musculoskeletal system, it is possible that some individuals had sub-clinical injuries that were not apparent at the time of enrollment. In the current study, variability in individual activity levels on the MBQ was considerable. According to these results, recreational activities ranged from no primary mode of exercise to running, biking, weight training and participating in group exercise classes. Habitual asymmetrical use of the limbs during daily function and recreation may create asymmetries in the tolerance of the neural tissues to movement. There is considerable evidence that habitual use of our limbs is not symmetrical during activities such as gait initiation [25], walking [26,27], turning [28], jumping [29-31], kicking [32], and crossing our legs [33]. While 85% of participants in the present study were right hand dominant (for writing) which is similar to proportions presented in previous literature, [34] a limitation to the present study is that lower limb dominance was not characterized in these individuals. Previous literature using various methods for determining limb dominance has shown a strong association between being right hand dominant and being right foot dominant (75.5%-93.5%), with a slightly lower association between left hand and foot dominance (56.9-79.4%) [34-36]. Lower limb dominance may have influenced the magnitude and direction of inter-limb asymmetries found in this study and further research is necessary to characterize the specific effects of lower limb dominance and asymmetrical activities on SLR range of motion.

Additional limitations include the small number of male participants, as equal distribution of men and women were not sought in this sample of convenience. It should be noted that the impact of sex that has been demonstrated in previous studies [23] was still evident in the present study despite unequal numbers of males and females. We did not account for the menstrual cycle in women participants, nor did we have participants perform a warm up prior to testing which are additional limitations to the present study, although it is hypothesized that the effect on SLR range of motion would be equal bilaterally and thus not affect inter-limb differences. Additionally, the high reliability demonstrated in the present study is limited to intra-rater, intra-session and cannot be extrapolated to comparisons between raters or between sessions measurements. Lastly, it is possible that small but clinically relevant correlations exist between demographic characteristics and range of motion measures that we were unable to detect due to inadequate power of the present study to detect correlations of 0.35 or less.

## Conclusion

Overall SLR neurodynamic testing range of motion is quite variable and tends to be greater in women, in those that are more active and in those that weigh less with a lower BMI. Inter-limb differences should be expected during SLR testing in healthy, asymptomatic individuals, but these asymmetries do not seem to be affected by the same demographic characteristics that influence overall SLR range of motion. Inter-limb differences of 11° or greater are outside of the normal range and thus may be valuable for comparisons to patients experiencing unilateral pain.

## Abbreviations

BMI: Body mass index (kg/m^2^); SLR: Straight leg raise; PF/SLR: Straight leg raise performed with the ankle in plantar flexion; DF/SLR: Straight leg raise performed with the ankle in dorsiflexion; MBQ: Modified Baecke Questionnaire; VPT: Vibration perception threshold; SD: Standard deviation; CI: Confidence interval; ICC: Intraclass correlation coefficient.

## Competing interests

The authors declare that they have no competing interests and verify that there is no financial affiliation with any product presented in this manuscript.

## Authors’ contributions

BSB conceived, designed and implemented the study and contributed to writing the manuscript. PSV implemented the study, assisted with analysis and contributed to writing the manuscript. Both authors read and approved the final manuscript.

## Pre-publication history

The pre-publication history for this paper can be accessed here:

http://www.biomedcentral.com/1471-2474/13/245/prepub
